# Determination of Pneumococcal Serotypes in Meningitis Cases in Niger, 2003–2011

**DOI:** 10.1371/journal.pone.0060432

**Published:** 2013-03-28

**Authors:** Jean-Marc Collard, Abdel-kader Alio Sanda, Jean-François Jusot

**Affiliations:** Centre de Recherche Médicale et Sanitaire (CERMES), Niamey, Niger; School of Medicine, Univ. Complutense, Spain

## Abstract

**Background:**

The epidemiology of pneumococcal meningitis in the African ‘meningitis belt’ is poorly studied. In order to ensure an effective vaccination strategy and post-vaccination surveillance, we examined the serotype distribution patterns of pneumococcal meningitis in Niger over the period 2003–2011.

**Methods:**

Cerebrospinal fluid (CSF) samples were collected from different health facilities throughout Niger in the frame of the national microbiological surveillance of meningitis. Determination of the serotype of CSF positive for pneumococci was performed using a sequential multiplex PCR method (SM-PCR) adapted with a national algorithm in which 32 different serotypes were covered and grouped into eight consecutive PCR.

**Results:**

The SM-PCR assay could predict the Sp serotype for 779 CSF (88.7%), 98 CSF (11.3%) were not-typeable in our national-adapted algorithm. In total, 26 different serotypes were identified. Serotype 1 (n = 393) was the most prevalent and accounted for 45.3% of infections, followed by serogroups/serotypes 12F/(12A)/(44)/(46) (7.3%), 6/(6A/6B/6C/6D) (5.4%), 14 (5.2%), 5 (4.6%), 23F (4.2%), 45 (3.6%), 2 (3.1%), 18/(18A/18B/18C/18F) (2.9%) and 17 others serotypes with a prevalence of less than 2%. The proportion of serotype 1 in infants(<2 years old) represented only 4.3% of the cases affected by this serotype. In contrast, serotypes 5, 6, 14, 19A and 23F were only detected in very young children.

**Conclusions:**

The proportion of serotype 1 in the pneumococcal meningitis cases and the theoretical vaccine coverage across all age groups advocates for the introduction of a conjugate vaccine (PCV10 or 13) into the Expanded Programme on Immunization (EPI) in Niger. Post-vaccine introduction surveillance supported by molecular approaches will be essential to provide a comprehensive picture of the impact of the vaccine on the burden reduction of pneumococcal meningitis and on pneumococcal serotype distribution.

## Introduction

Historically, meningitis epidemics in sub-Saharan Africa have been due to *Neisseria meningitidis* (Nm) particularly serogroup A, responsible for the majority of the disease burden and accounting for several thousand cases every year. *Streptococcus pneumoniae* (Sp) was not supposed to occur in epidemics, however, pneumococcal meningitis (PM) epidemics have been well described in Senegal (serotype 5) in 1983 [1] and more recently in Ghana in 2005 (serotype 1) [2]. Recurrent PM epidemics, mainly due to serotype 1 strains, have been observed in Burkina Faso and Togo from 2006 [3], mirroring the seasonality of meningococcal meningitis.

The introduction of the MenAfriVac™ vaccine (conjugate vaccine against serogroup A meningococci) in Burkina Faso, Mali and Niger has already contributed to a substantial decline in the number of meningitis cases due to meningococci irrespective of the ability of currently circulating serogroups W [4] and X [5] to lead to small-scale epidemics. The decline of meningococcal meningitis next to the MenAfriVac™ campaign was concomitant to the rise PM cases, mainly in Burkina Faso. Globally, from 2009 to 2011, the absolute number and the proportion of Sp cases (%) in positive, non-contaminated CSF collected in the African meningitis belt rose from 358 to 879 and from 12.8 to 47.6, respectively [6].

In the countries of the meningitis belt, case fatality rates (CFR) associated with pneumococcal meningitis generally exceeded those of meningococcal meningitis and can be as high as 50% [3], [7], [8]. During the last decade though, there has been the significant opportunity to reduce the burden of invasive pneumococcal disease through the use of a conjugate vaccine. An ever-increasing number of countries have applied for vaccine funding by GAVI Alliance which has recently announced its support of 17 additional countries (including 14 in Africa) to introduce conjugate pneumococcal vaccines [9]. The roll out for Niger, a sub-Saharan country of 1,267,000 square kilometers, with an estimated population of 15 730 754 inhabitants in 2011 of which 49.2% under 15 years old, is expected to start in 2014.

Currently, over 90 pneumococcal serotypes are recognized and approximately a quarter of these serotypes are responsible for the majority of cases of invasive pneumococcal disease [10]. In developed countries, serotype distribution of Sp is still monitored by culture of the organism from biological samples followed by serological determination of the capsular type by the standard capsular test (gold standard), such as Quellung reaction. However, a first PCR-based serotyping assay was developed by Pai and colleagues in the United States in 2006 [11] and this sequential multiplex PCR (SM-PCR) assay is being increasingly used due to its potential to overcome some of the difficulties associated with serologic testing and offers the opportunity to be adapted to specific geographical areas [12]–[17]. In low resource countries, such Niger, the PCR based assay for direct detection of select serotypes from clinical specimens is a valuable tool to circumvent the serious drawbacks of the gold standard system such as the difficulty associated with culturing specimens in very remote areas, the high costs of reagents and the level of technical expertise required to perform the Quellung reaction.

In this study, the serotype distribution pneumococci responsible for meningitis from 2003 to 2011 was investigated to predict the potential coverage of the 10 or 13-valent pneumococcal conjugate vaccine (PCV10 or PCV13) scheduled for introduction in the Nigerien vaccination programme in 2014 and to assess the potential for serotype replacement post vaccine introduction.

## Methods

### Microbiological surveillance of meningitis and collection of CSF

Meningitis surveillance in Niger is undertaken by the Direction of Surveillance and Response to Epidemics (DSRE), Ministry of Public Health (MoPH). Quantitative data on the morbidity and the mortality by age group for all clinically suspected cases of meningitis meeting a standard case definition are notified on a weekly basis. This surveillance is coupled to the cerebrospinal fluid (CSF) microbiological surveillance across Niger performed by the Centre de Recherche Médicale et Sanitaire (CERMES) based in Niamey. Aliquots of CSF samples were collected daily during the epidemic season from all national hospitals within Niamey. Additionally, between November and June each year from 2003–2010, a monthly collection of clinically well-defined samples occurred from hospitals/health facilities within a radius of 300 km from Niamey (Dosso and Tillabéry Regions) by CERMES. Frozen or refrigerated CSF samples from the rest of the country were sent on a voluntary basis to CERMES or DSRE by mandated transport companies.

### Bacteriological and molecular analysis of CSF

Laboratory confirmation of bacterial meningitis was performed by CERMES using culture and/or PCR techniques on CSF or CSF-inoculated trans-isolates (TI) as previously described [18]. Briefly, after a cycle of freezing (−20°C) and thawing (5 min at 100°C), CSF samples (200 to 500 µl) were centrifuged and the DNA-containing supernatants were collected for PCR testing. Conventional multiplex PCR was performed to detect the presence of *N. meningitidis* (*crgA* gene), *S. pneumoniae* (*lytA* gene) and *H. influenzae* (*bexA* gene) in the conditions described by Chanteau *et al*. [19].

Positive CSF detected by the multiplex PCR targeting the *lytA* gene were retained for typing.

Determination of the serotype of pneumococci in CSF was performed using the SM-PCR method initially described by Pai *et al.* [11] and expanded by the CDC to include up to 40 serotypes/small serogroups [12]. However the SM-PCR used in this study was adapted with a national algorithm covering 32 different serotypes grouped into eight PCRs (Table 1). This adaptation was based on the distribution of 86 pneumococcal strains isolated between 2003 and 2008 and serotyped by the use of the Quellung reaction (Hamidou AA, unpublished data) by the National Reference Center for Pneumococci–NRCP-(Hôpital Pompidou, Paris, France).

**Table 1:** Mutiplex design.

	Primers	Amplified serotypes	Amplicon size (pb)	Primer concentration (µM)	References
Reaction 1	24	24/(24A, 24B, 24F)	99	2	[12], [20]
	1	1	280	1.5	[11], [12]
	23F	23F	376	1.5	[11], [12]
	18	18/(18A/18B/18C/18F)	576	2	[11], [12]
Reaction 2	14	14	189	1.5	[12], [14]
	12F	12F/(12A/44/46)	238	1.5	[11], [12]
	45	45	376	1.5	[12], [16]
	35F	35F/47F	517	2	[11], [12]
Reaction 3	48	48	99	2	[21]
	6	6/(6A/6B/6C/6D)	250	1.5	[11], [12]
	5	5	362	1.5	[11], [12]
	4	4	430	1.5	[11], [12]
Reaction 4	25F	25F/38	280	1.5	[21]
	2	2	381	1.5	[16]
	19A	19A	566	1.5	[12], [22]
	9V	9V/9A	816	2	[12], [20]
Reaction 5	39	39	98	1.5	[12], [20]
	10F	10F/(10C/33C)	248	1.5	[12], [20]
	12B	12A/12B/12F/44/46	350	1.5	[23]
	20	20	514	1.5	[11], [12]
Reaction 6	8	8/39	98	1.5	[12], [20]
	15B	15B/15C	248	1.5	[11], [12]
	9N	9N/9L	350	1.5	[12], [14]
	35B	35B	677	1.5	[11], [12]
Reaction 7	7C	7C/(7B/40)	260	1.5	[11], [12]
	19F	19F	303	1.5	[11], [12]
	38	38/25F/25A	574	1.5	[11], [12]
	22F	22F/22A	643	1.5	[11], [12]
Reaction 8	3	3	280	1.5	[11], [12]
	33F	33F/(33A/37)	338	1.5	[11], [12]
	10A	10A	566	1.5	[11], [12]
	35A	35A/(35C/42)	816	1.5	[12], [20]

All PCR primer pairs were validated by multiplex PCR reactions against DNA from known serotyped strains obtained from the Biological Resource Center of the Pasteur Institute (Paris, France) or isolated in Niger and serotyped by the NRCP.

All *lytA* positive CSF were first screened with a *cpsA* PCR amplification and *cpsA* positive CSF were subsequently analysed in the sequential PCRs all including four primer pairs for different serotypes. Remaining *lytA* positive/*cpsA* negative CSF were screened in simplex PCR for serotypes 25F and 38.

### Data management and analysis

Statistical analysis with the open-source R2.14.0. (2011) software (R Foundation for Statistical Computing, Vienna, Austria) were used to describe the epidemiology of pneumococcal meningitis using both data from epidemiological surveillance ensured by MoPH and from microbiological surveillance performed by CERMES.

### Ethics statement

All specimens (CSF) were collected by clinicians with patient or family oral consent after being informed why their CSF was sampled. This was done as part of the routine clinical management of patients according to the national guidelines in Niger. By no means, additional CSF sampling for research purpose was done in the frame of this study. Remaining aliquots of CSF after routine CSF examination by health facilities for case management were sent on a voluntary basis to CERMES to meet enhanced microbiological surveillance of meningitis guidelines. CERMES is a Technical and Scientific National Institution from the Ministry of Public Health (Decree n° 2005-060/PRN/MSP/LCE) with a Board of Directors in which several representatives of the Ministry, as well as a representative of the National Ethics Committee, are sitting. CERMES was designated and committed by a decree from the Ministry of Public Health as National Reference Laboratory (NRL) for meningitis in Niger (249/MSP/DGSP/DPHL/MT). Furthermore, an official written consent of the Ministry of Public Health was obtained for collection and microbiological analysis of CSF (2011/000699/MSP/DGSP). Confidentiality on patients' identity was also guaranteed. As such, written consent was not sought and approval from the national ethics committee was not required.

## Results

### Surveillance data and CSF collection

From 1^st^ January 2003 to 31^st^ December 2011, the MoPH of the Republic of Niger reported a total of 42,013 suspected cases of meningitis and 19,223 CSF were collected and analysed (45.8%) at CERMES. Molecular analyses of all CSF samples obtained from 2003 to 2011 revealed 1010/19,223 CSF positive results by *lytA* PCR: 5.3% of all CSF, 12.6% of those having a known aetiology (*crgA*, *lytA* and *bexA* positive; N = 8041). Among those 1010 *lytA* positive CSF, 86 were either missing or the CSF volume was depleted. All other CSF (n = 924) were screened again with a simplex PCR for *lytA* and *cpsA* genes. A total of 80 CSF were negative for *cpsA* (57 were *lytA* negative, *cpsA* negative and 23 were *lytA* positive, *cpsA* negative).The 57 *lytA* negative, *cpsA* negative CSF were not included in the SM-PCR but the 23 *lytA* positive, *cpsA* negative CSF were analysed separately in simplex PCR for serotypes 25F and 38. The years recording the highest recovery rate of Sp were 2004, 2005 and 2007 with 26.1, 37.7 and 36.4% of all CSF positive for a known aetiology (either *crgA*, *lytA* or *bexA* positive), respectively.

### Serotype determination

Our adapted SM-PCR covering 32 different serotypes provided the Sp serotype for 779 CSF (88.7%), with 98 CSF (11.3%) remaining not-typeable (SM-PCR NT). In total, 26 different serotypes were identified (Table 2). Serotype 1 (n = 393) was the most prevalent and accounted for 45.3% of all infections, followed by serotypes 12F/(12A)/(44)/(46) (7.3%), 6/(6A/6B/6C/6D) (5.4%), 14 (5.2%), 5 (4.6%), 23F (4.2%), 45 (3.6%), 2 (3.1%),18/(18A/18B/18C/18F) (2.9%).

**Table 2:** Serotype proportion by SM-PCR in PM cases in Niger, 2003–2011.

Serotype by SM-PCR	Number of cases	Percentage
1	393	45.3
12F/(12A)/(44)/(46)	63	7.3
6/(6A/6B/6C/6D)	47	5.4
14	45	5.2
5	40	4.6
23F	36	4.2
45	31	3.6
2	27	3.1
18/(18A/18B/18C/18F)	25	2.9
38/25F/25A	14	1.6
25F/38	6	0.7
10F/(10C/33C)	6	0.7
19A	5	0.6
12B/(12A/12B/12F/44/46)	5	0.6
9V/9A	5	0.6
24/(24A, 24B, 24F)	5	0.6
8	4	0.5
35B	2	0.2
9N/9L	2	0.2
39	2	0.2
15B/15C	1	0.1
33F/(33A/37)	1	0.1
35F/47F	1	0.1
7C/(7B/40)	1	0.1
20	1	0.1
48	1	0.1
SM-PCR NT	98	11.3
**Total**	**867**	**100.0**

The 1^st^ PCR reaction was able to detect the serotype in 53.6% of all positive CSF tested. Reactions 1 and 2 covered 69.1%, reactions 1–3 79.2% and reactions 1–4 84.2%. Serotype 1 represented 59.3% of all positive CSF in 2003. Serotypes 14, and 18 were not detected in 2003, serotype 19A was not detected in 2003, 2008, 2010 and 2011 and serotype 2 was not detected in 2011. On the 23 *lytA* positive, *cpsA* negative CSF, 13 were positive for the serotype 38 but all negative for serotype 25F. These additional analyses rose the % of serotype 38 from 0.1 to 1.63%. The 10 remaining negative CSF were included in the SM-PCR NT CSF.

### Serotype distribution in age groups and in regions

**Figure 1:**
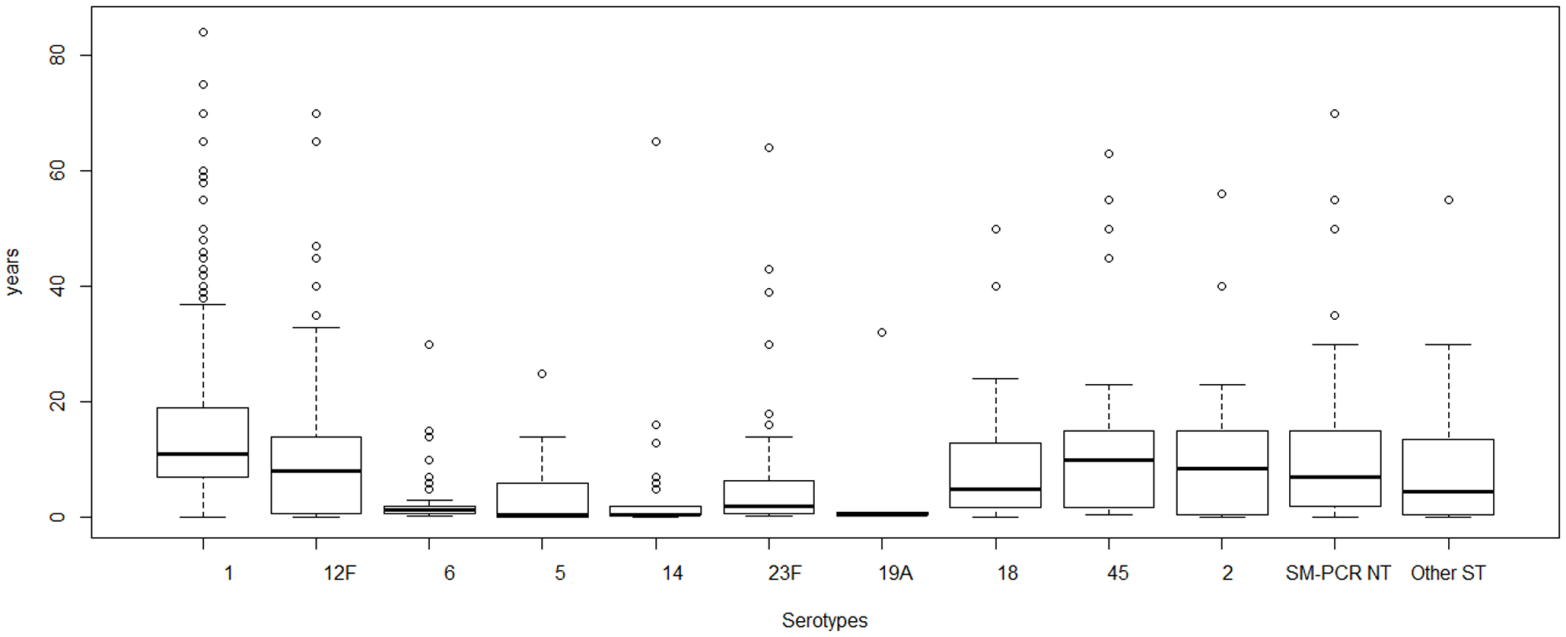
Age distribution for the prevalent serotypes. Box plots representing age according to serotype. Box plots provide distribution quartiles of 25%, 50%, 75% and 100%; Box length indicates interquartile ranges. Circles: outlier values; thick horizontal lines: median values; whiskers: range of values within 1.5 interquartile range.

**Figure 2:**
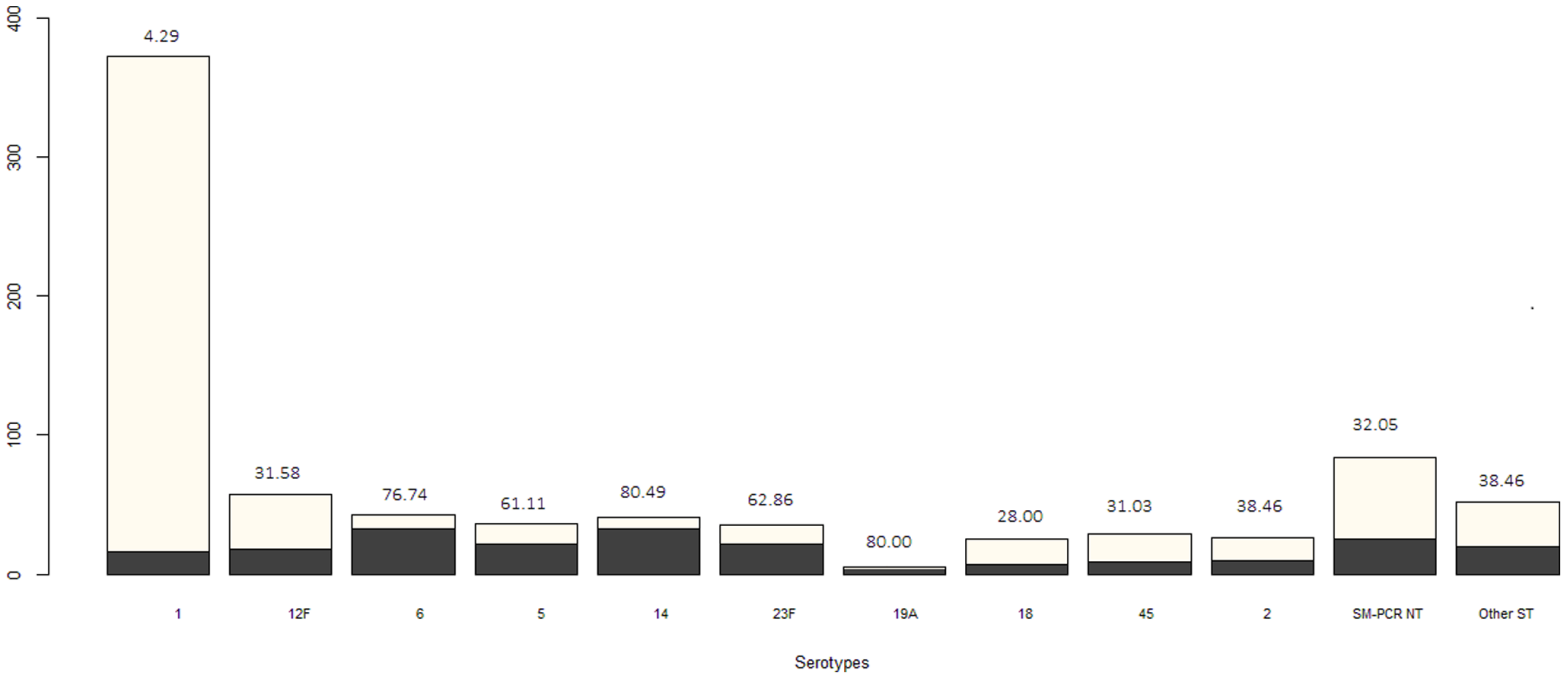
Number of PM cases in under 2 year olds (black) and over (grey) 2 years old for the prevalent serotypes. Percentages of cases in children <2 years are given over the bar plots.

Serotype 1 was detected primarily amongst older children, teenagers and young adults (5 to 20 years old) (Figure 1). The proportion of serotype 1 in infants(<2 years) represented only 4.3% of the cases affected by this serotype (Figure 2). Serotypes 2, 12F and 45 were encountered in the same age groups as serotype 1. In contrast, serotypes 5, 6, 14, 19A and 23F were only detected in very young children. The proportion of serotypes 5, 6, 14, 19A and 23F in infants(<2 years) represented 61.1%, 76.7%, 80.5%, 80.0% and 62.9% of the cases, respectively (Figure 2).

**Figure 3:**
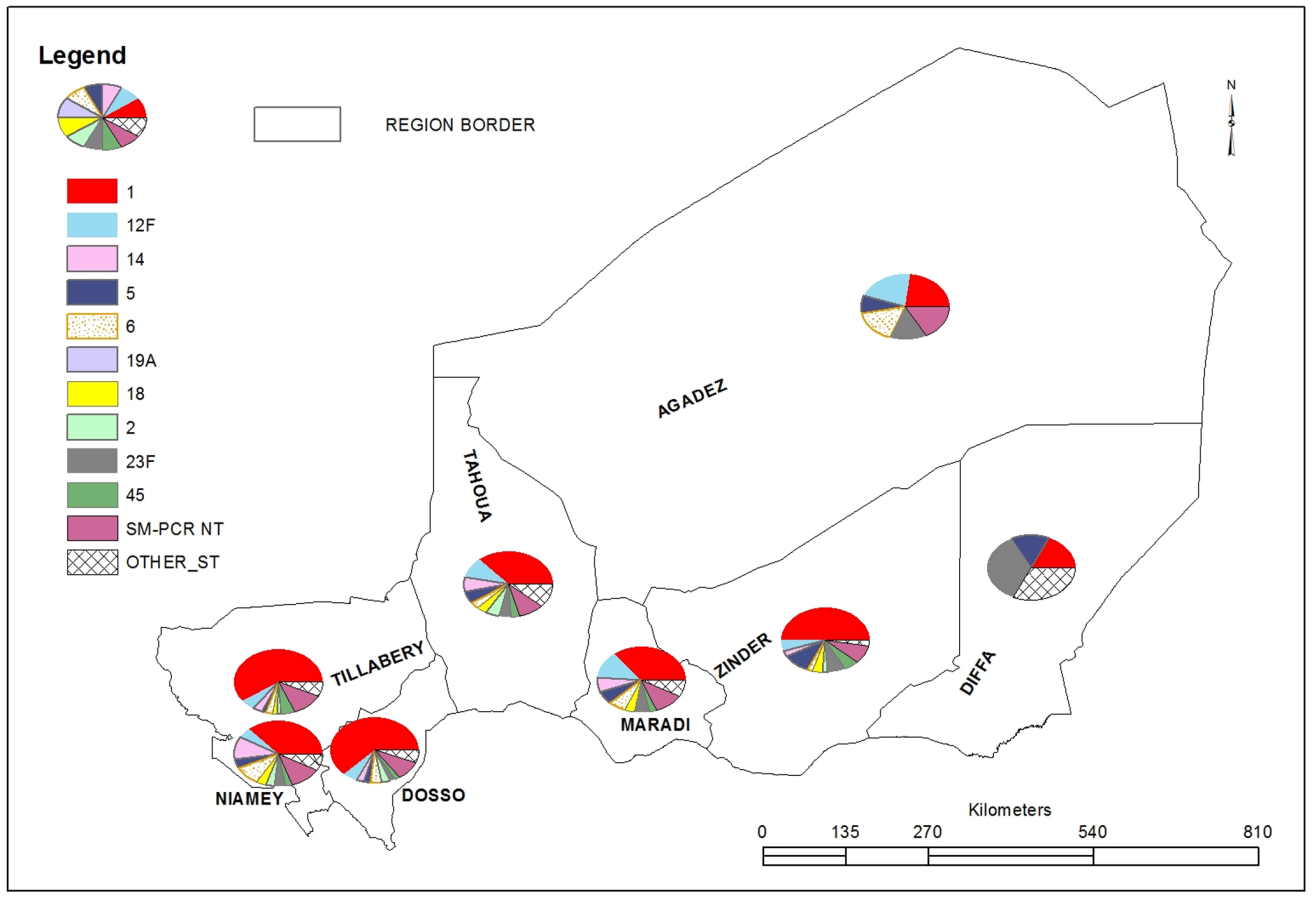
PM serotype distribution by Region, 2003–2011.

Figure 3 shows the serotype distribution in Regions for pneumococcal cases across Niger, when the geographical origin of the patient was known (99.4%). Serotype 1 was predominantly found in Western Niger, particularly in Tillabery and Dosso Regions with more than 58.9% of cases, and to a lesser extent in Niamey (38.0%). Serotype 12F was predominant in the Maradi and Agadez regions.

## Discussion

Accurate serotype determination of *S. pneumoniae* from meningitis cases is essential for accurate epidemiological surveillance in the context of vaccine introduction.

The rollout of pneumococcal vaccines in the developing world through the considerable support of the GAVI alliance is now underway across three continents and Niger is one of the eligible countries for support in 2014. This paper presents the most comprehensive data to date detailing the national baseline serotype distribution for Niger as a basis to assess the potential coverage and future impact of the pneumococcal-conjugated vaccine selected for introduction. However there were, as with any long term surveillance programme, some limitations to our data. The national meningitis surveillance system, relies on biological samples (mainly CSF) received by the National Reference Laboratory which are either refrigerated or frozen. Yet as a consequence of poor infrastructure at peripheral laboratories (regional or district level of the sanitary pyramid) most of the samples were not viable on arrival. CSF recently withdrawn and kept at room temperature or seeded in Trans-isolates allowed the culture of aetiological agents but samples of this type were rare from sentinel sites. As such molecular typing is the only workable option to determine the serotype of Sp in CSF in our context. Although a suitable, adaptable and increasing used method, the simple and schematic sequence-based system of sequential multiplex PCR (SM-PCR) has some disadvantages. The main disadvantage is its inability to discriminate closely related serotypes, however some improvements have been made to circumvent this [23], [25]. One of the first reports describing the use of a reformulated PCR algorithm for serotyping PM directly on CSF was published by Lafourcade *et al.* [17] with a sensitivity and specificity of 80 and 100%, respectively. This multiplex PCR assay required little technical skills and is implementable in any country with basic PCR capabilities.

Our Niger adapted SM-PCR was able to determine the Sp serotype in 88.7% of *lytA* positive CSF from PM and in total, 26 different serotypes were identified between 2003–2011. Supplementary PCR amplification on 23 *lytA* positive, *cpsA* negative CSF revealed 13 cases of serotype 38 bringing its representativity to 1.63%. This phenomenon has been observed previously with serotypes 38 and 25F generally negative for *cpsA* [20], [25] and so was not unexpected. Over the study period, serotype 1 was the most common serotype (45.3%) and accounted for 35.1% (2007) to 59.3% (2003) in PM cases investigated. Globally, serotype 1 is ranked in the top 4 serotypes across every region, with the exception of North America and Oceania [26]. A recent marked increase in the prevalence of serotype 1 disease in Europe was noted firstly in Sweden and subsequently in other countries including England, Wales, Scotland and Portugal [27]. In a systematic review of literature from 1970 to 2010 done by Gessner *et al.* [8], it was shown that, in Western Africa, after 5 years of age, 59–79% of all meningitis cases (depending on the country-Burkina Faso, Ghana and Senegal) were due to serotype 1. Serotype 12F, the second ranked serotype in Niger (7.3%), has also been reported in 10% of Sp invasive cases from Burkina Faso [17] but interestingly not in Algeria [28], even though both countries share borders with Niger but Algeria and Niger are separated by a desert (Ténéré) very sparsely populated. Serotype 12F was also found in IPD cases in the United States (4.9%) [11], Seville in Spain (6.45% in adult patients; 0% in paediatric patients) [29], Bangladesh (7.8%) [16], Mozambique (0.65%) [15] and Malawi (6%) [30], but not in Brazil [14]. Nevertheless, given its prevalence in different countries, this serotype was not included in any pneumococcal polysaccharide or conjugated vaccine formulations.

**Figure 4:**
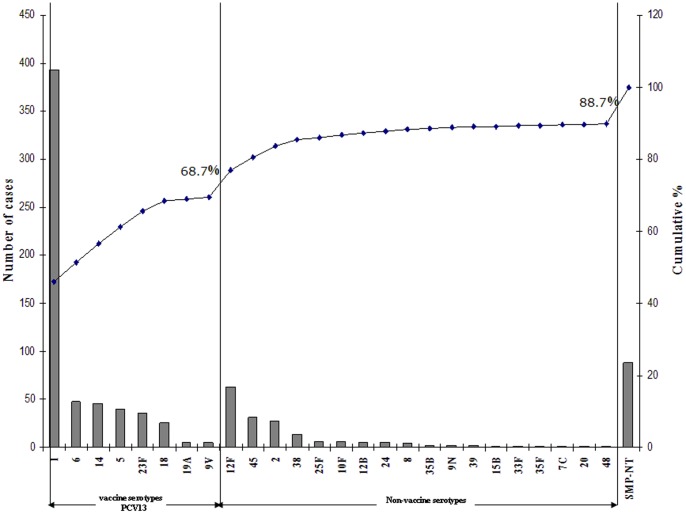
Bar chart showing rank order and cumulative serotype distribution (%) for PCV13 (left) and non PCV13 (right) serotypes.

If we consider the coverage of PCV10 across all age groups, 68.2% of circulating serotypes in Niger would be covered and this includes all serotypes frequently associated with infants in our setting with the exception of 19A. This serotype is included in PCV13 and so coverage would increase to 68.7% (Figure 4). However, serotype 12F, representing 7.3% of the PM cases (2^nd^ most predominant circulating serotype) is not included in any of the conjugate vaccines.

Conjugate vaccines have been generally recommended for routine use in neonates and children under than 2 years of age whereas the pneumococcal polysaccharide vaccine (PPV) is generally recommended for all adults 65 years and older and for 5 to 64 year olds who have a chronic disease or illness, an impaired immune system, or who live in areas or among social groups where there is an increased risk for pneumonia or meningitis. Our results showed that serotype 1 (representing 45.3% of all cases) is more commonly identified in children aged >5 year, in teenagers and young adults (5 to 20 years old). The proportion of serotype 1 in those under 2 years old, represented just 4.3% of all serotype 1 cases. Although they are often found in disease, serotype 1 pneumococci are seldom carried or are simply hard to detect in carriage studies due to technical reasons. If carried in very young children, the introduction of the conjugated vaccine in the Expanded Programme on Immunization (EPI) could induced a herd immunity effect and consequently reduce the burden encountered in older children and young adults. On the other side, the proportion of other serotypes such as 5, 6, 14, 19A and 23F in under 2 years old children represented 61.1%, 76.7%, 80.5%, 80.0% and 62.9% of cases respectively. These data would therefore support the introduction of a conjugate vaccine (PCV10 or PCV-13) into the EPI. In the future it would be beneficial to see the development or expansion of formulations which are of relevance to Africa to provide greater coverage in an area suffering from the highest burden.

## Conclusions

Accurate serotype determination of *Streptococcus pneumoniae* involved in meningitis cases is essential in the context of vaccine introduction and epidemiological surveillance. Our study represents the first comprehensive report outlining the national baseline serotype distribution of PM in Niger prior to the introduction of a pneumococcal conjugate vaccine in 2014.

Across the study period (2003–2011), serotype 1 was the most commonly identified serotype and accounted from 35.1% to 59.3% of all CSF investigated dependent on year. This report also underlines the importance of having sustained and high quality microbiological surveillance of PM complemented by accurate analytical methodologies for determining pneumococcal serotypes in Niger.
